# Positive Medical Agents

**Published:** 1855-03

**Authors:** 


**POSITIVE MEDICAL AGENTS**; Being a Treatise on the New Alkaloid, Resinoid and Concentrated Preparations of Indigenous and Foreign Plants. Published, by Authority of the American Chemical Institute.

A late number of the American Medical Gazette, edited by Dr. Reese, of New York, contains a favorable notice of this book.

The object of the work seems to be to call the attention of physicians to some of those preparations, usually denominated the "active principles," of certain plants known to possess medicinal properties. Doubtless there are many valuable medicines belonging to the vegetable kingdom which would be more widely used, were it not for the bulk of the required doses and the uncertainty of their action. It is certainly a desideratum with every physician to obtain reliable remedies, as well as to be able to diminish the doses to a take-able size. These two objects are apparently accomplished in the use of the "active principles." The number of such preparations, already contained in the list of standard medicines, and published in our Dispensatory and the other works of Materia Medica, is considerable. This fact, the author of the book before us, notices and gives a brief account of some of the more prominent, though he devotes a great majority of his pages to the consideration of some which are of more recent discovery. Most of the remedies of which he treats, have been used to a greater or less extent in the crude state, by physicians. Abundance of testimony as to the value of these medicines may be found in various medical writings.

If those, whose virtues have been well attested, can, by aid of the pharmaceutist, be reduced to an uniform standard of strength and reliability, a great service will thus be rendered to our science.

We regret that the author does not give formulæ for preparing the different medicines of which he treats. We hope to see this deficiency supplied in a future edition.

The account of the medicinal effects of the substances considered, has been derived we are told, from experience and careful observation. The mode of using them, together with the manner in which they may be advantageously combined is described, and these two points are illustrated by reports of cases actually treated by these agents.

The entire work evinces the author's decided partiality for the "Positive Agents" of the vegetable portion of the Materia Medica; yet its tone is good, and the spirit in which it is written will commend its pages to every candid, unprejudiced mind.

The duty of the physician when called upon to treat disease, generally compels him to make use of the most positive remedial agents at his command; and of the native properties and effects of these, whether drawn from the mineral or vegetable kingdoms, he must be well informed. We know that many have conscientiously abandoned certain valuable vegetable remedies, simply because they could not be depended on for uniformity and strength of action.

The agents which are considered in the above treatise, are prepared and sold by B. Keith & Co., 582, Houston-st., New York, who "warrant them pure, in all cases."

